# A Question of Propriety

**Published:** 1886-03

**Authors:** 


### A QUESTION OF PROPRIETY.

There seems to be a misunderstanding of the common ethics of journalism by some contributors. An address, an essay or an article has a distinct value, and its ownership must be vested in some one. Until otherwise disposed of this rests with the author, but when by purchase or free gift he has conveyed it to another, it is as clearly the property of the devisee as would be any other possession. If it be conveyed to a professional or other journal the original author has no longer control of it, but it is absolutely the property of that journal, and its surreptitious acquirement and use by another is not justified by any rule of right.

An article cannot be published as "original" by but one journal, unless there is a clearly understood partnership in its publication between two or more. If an article be conveyed to one journal as "original," and the author at the same time sends copies to other journals without giving the first one due notice, he has violated all the rules of journalistic and common honesty, for he has sold the same property to two or more people. It matters not whether there be a money consideration, for the expense of putting an article in type gives the publisher a monied interest in it, and the author may accept for his compensation the wide dissemination of his views.

It is a matter of common courtesy among publishers to allow the republication of an article when the proper credit to the rightful owner is given, but this is clearly a matter of courtesy, for the ownership rests with the original proprietor. It is a literary theft when an article is republished without acknowledgment of the rightful ownership. This journal seldom has occasion to republish from other journals, because it usually has sufficient matter of its own. These is a deal of borrowing from our pages, the honorable journals among our exchanges always giving the proper credit, but there are a few thieves, some at home and some abroad, with whom in due time we shall have a reckoning. When we borrow matter from another journal we shall always be careful to give proper credit.

This assertion demands an explanation of the appearance in our last month's issue of the admirable address by Dr. Geo. L. Parmele, subsequent to its publication in *The Archives of Dentistry*. The editor of this journal was present at the reading of that address, and it was there and then given to him for publication. The Secretary of the society requested that it be returned him after it was in type, that he might make an abstract of it for his minutes, and this was done. The article was crowded out of our January number, and to our astonishment it appeared in the January number of *Archives*, which did not reach our table until about the twentieth of the month, after the first form of our February number, which contained the article, was all printed. We at once wrote Dr. Parmele to know how this occurred, and were assured by him that our letter gave him the first intimation of its publication. He said that he was not ignorant of the ethics of journalism, and the publication was entirely without his knowledge or consent, and markedly contrary to his wishes. He had furnished no copy for such publication, nor had he ever made a second transcript of the article. He had given it to The Independent Practitioner, as he had a right to do, and if another had obtained possession of it he was not a party to it. He desired that a public expression of this be given, that he might not rest under the onus of the transaction.

We do not desire to be understood as intimating that the editor of *Archives* was in any way privy to this unwarranted publication. We have reason to believe that the article was published in good faith, and without knowledge that it was the property of another journal. He probably received it from one who was supposed to have the right to transfer it, and in that case is blameless. We write this explanatory article that neither Dr. Parmele nor the editor of THE INDEPENDENT PRACTITIONER may be held responsible for this breach of journalistic etiquette, but that the blame may be placed where it properly belongs. Hereafter we shall exercise a little more caution in permitting manuscript to go out of our hands before the day of publication.

